# Longer-term experiences of families of children with dextro-transposition of the great arteries: a qualitative study

**DOI:** 10.1038/s41390-025-04201-y

**Published:** 2025-07-17

**Authors:** Karen J. Eagleson, Theresa I. Chin, Stephen Larmar, Robert N. Justo, Nadine A. Kasparian, Samudragupta Bora

**Affiliations:** 1 Faculty of Health, Medicine and Behavioural Sciences, The University of Queensland, Brisbane, QLD Australia; 2 Queensland Paediatric Cardiac Service, Queensland Children’s Hospital, Brisbane, QLD Australia; 3 Department of Pediatrics, Brigham and Women’s Hospital, Harvard Medical School, Boston, MA USA; 4 Division of Biology and Medicine, The Warren Alpert Medical School of Brown University, Providence, RI USA; 5 School of Human Services and Social Work, Griffith University, Brisbane, QLD Australia; 6 Heart and Mind Wellbeing Center, Heart Institute and Division of Behavioral Medicine and Clinical Psychology, Cincinnati Children’s Hospital Medical Center; Department of Pediatrics, University of Cincinnati College of Medicine, Cincinnati, OH USA; 7 Health Services Research Center, University Hospitals Research & Education Institute; Department of Pediatrics, University Hospitals Rainbow Babies & Children’s Hospital, Case Western Reserve University School of Medicine, Cleveland, OH USA

## Abstract

**Background:**

Explore longer-term psychosocial adaptation to congenital heart disease among families of children with dextro-Transposition of the Great Arteries (d-TGA).

**Methods:**

In this qualitative study, semi-structured interviews were conducted for 16 families (16 mothers, 12 fathers, 9 siblings) and analyzed using an inductive thematic approach.

**Results:**

Key qualitative themes included parents reflecting “back then” when 1) navigating hospital and healthcare experiences, 2) challenges to psychosocial wellbeing and family functioning, and 3) coping and support needs were greatest. Parents described 4) key transitions and defining moments, and 5) feeling “lucky” and grateful for contemporary cardiac care and when compared to families of children with univentricular conditions and valuing a “normal” life. Furthermore, 6) the surgical scar served as a constant reminder of past experiences and 7) uncertainty about the role of the cardiac condition in their child’s development persisted for some parents. Key themes among siblings included 1) having a “normal” family life, 2) positive and negative aspects of the sibling relationship, and 3) limitations in understanding their sibling’s cardiac condition.

**Conclusion:**

Families of children with d-TGA value “normal” family lives years after surgical intervention. To improve care and support, a focus on positive psychosocial adaptation, including individual and family resilience, parental perceptions of surgical scars, and self-reported sibling experiences, is required.

**Impact:**

- Families of children with complex congenital heart disease are at risk for psychosocial and family functioning difficulties. Research has focused on early experiences of diagnosis and hospitalization, with limited evidence of longer-term experiences.
- Using qualitative methodology, we explored the lived experiences of families of children with dextro-Transposition of the Great Arteries concerning longer-term psychosocial adaptation to congenital heart disease.
- Despite ongoing illness uncertainty and reminders of previous medical experiences, parents and siblings of children with dextro-Transposition of the Great Arteries predominantly described positive adaptation and living and valuing their “normal” family lives years after arterial switch operation.

## Introduction

Infants with dextro-Transposition of the Great Arteries (d-TGA), a complex cyanotic congenital heart disease (CHD), require early open-heart surgery. Long-term survival is high, and freedom from re-operation after arterial switch operation (ASO) is common.^1^ Nonetheless, lifelong specialized cardiac care is required, with a heightened risk for neurodevelopmental and psychological difficulties^2–4^ and subsequent increased use of developmental services.^5–8^

While longer-term outcomes of children with d-TGA have been documented, fewer studies have focused on their families’ experiences.^9,10^ Increased risk of psychological distress has been reported in parents of children with complex CHD,^11–13^ with both persisting^14,15^ and decreasing levels^10,16–18^ over time, as well as both negatively impacted family functioning^19,20^ and greater family cohesion.^21,22^ In parents of children with d-TGA, significantly less parenting stress and more social support than normative samples have been reported at both 1 and 4 years of age.^10^ Compared with families of young children with hypoplastic left heart syndrome (HLHS),^9^ parents of children with d-TGA reported lower negative family impact, with increased closeness because of their child’s medical experiences, reported by 96% of families.

Qualitative research enables the exploration of participants’ lived experiences and perspectives.^23^ To date, no qualitative studies have focused specifically on the experiences of families of children with d-TGA. With a predominantly homogeneous clinical course, qualitative exploration provides the opportunity for an improved understanding of the experiences of families of children with complex CHD.^24,25^ Furthermore, the number of qualitative studies inclusive of mothers and fathers beyond early hospitalization,^26^ and siblings overall, is very limited.^27^ Thus, our study aimed to explore the longer-term experiences of parents and siblings of children with d-TGA using a qualitative methodology, and informed by a family adaptation to chronic illness conceptual model.^28^

## Methods

### Participants

A contemporary cohort of families of all living children with d-TGA with Intact Ventricular Septum or Ventricular Septal Defect born between 1 January 2008 and 31 December 2017 who underwent ASO at the Queensland Paediatric Cardiac Service was established. Exclusion criteria were families of children with complex d-TGA unsuitable for ASO, with chromosomal, syndromic, or major non-cardiac comorbidities (excluding neurodevelopmental disorders) or born <37 weeks gestation; families with insufficient English language skills to participate without an interpreter; and siblings younger than the child with d-TGA or under age 8 years. A matrix was developed to facilitate purposive sampling with representation across three child age groupings, time of diagnosis (prenatal vs. postnatal), and family residential location (urban vs. regional).

### Procedures

This study was informed by the Consolidated Criteria for Reporting Qualitative Research guideline.^29^ Ethics approvals were obtained from Children’s Health Queensland (HREC/19/QCHQ/56798) and The University of Queensland (2019/HE002627) Human Research Ethics Committees. A semi-structured interview guide (Supplement 1) was developed by the multidisciplinary research team (psychology, pediatric cardiology, nursing), and refined based on piloting with parents (*n* = 2) of children with a cardiac and non-cardiac chronic condition.

Study information was mailed to families to consider their interest in participation. Families were contacted after two weeks to address questions and schedule interviews, either face-to-face in the family home or research center, or virtually via Zoom. Written informed consent was obtained from parents and adult sibling participants, and child assent and parental consent were obtained for sibling participants <18 years.

Interviews took place between February and June 2020. All interviews were conducted individually by the first author (advanced practice pediatric cardiac nurse; female) and audio-recorded with permission. The first author’s dual role as researcher and clinician (including previous care of some participants) was explicitly discussed with families to promote clear boundaries and enable “data collection vs. therapeutic interaction”.^30^ Three early interviews (five participants) were attended by another author with expertise in qualitative research methodology (SL; male). To support wellbeing, time was spent with participants post-interview, with additional follow-up after approximately one month.

### Data analysis

Our sample size was informed by recommendations concerning thematic analysis, informational saturation, and sampling methodologies.^31,32^ Informed by Miles et al.^33^ an inductive and iterative approach to qualitative data analysis was used. Interview recordings were transcribed verbatim,^23^ with three parent transcriptions reviewed for accuracy by another author (TC; female). First-cycle codes were created, and codebooks were established, with subsequent second-cycle coding (parent interviews only) to organize codes into early themes. An example of theme development is provided in Supplement 2. One-third (*n* = 12) of transcripts were coded by two authors (KE, TC) with agreement reached through discussion, and selected early parent interview transcripts (*n* = 2) were independently reviewed by two additional authors (SL, NK) who are experienced coders. Regular discussions occurred within the research team through all stages of analysis, with records kept. NVivo 12 software (Lumivero, Denver, CO) was used to organize coding, theme development, annotations, and reflexive memos. Microsoft Excel was used to develop matrices/summaries to enable comparisons between family members’ responses across themes.

## Results

**Fig. 1:**
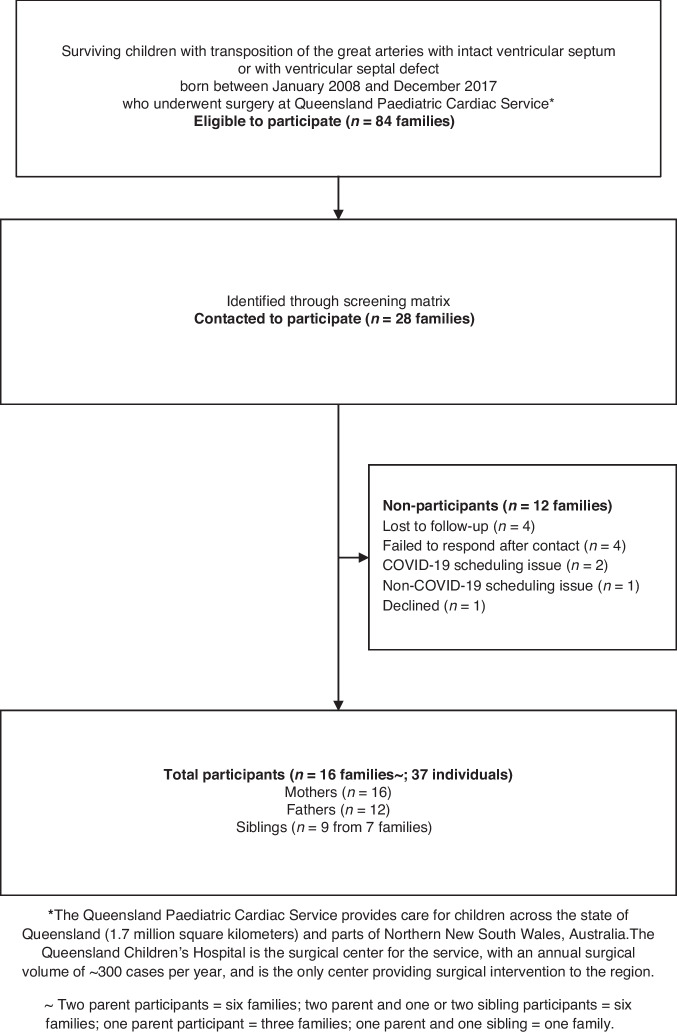
Participant recruitment flow diagram. This visual representation illustrates the procedure for recruiting study participants and the reasons for non-participation.

Of the 28 families approached, 16 participated (57% response), with 37 participants (16 mothers, 12 fathers, and 9 siblings) interviewed (see Fig. 1). Clinical and demographic data are shown in Table 1. The mean age of children with d-TGA was 6 ± 3 years, 56% were diagnosed prenatally, and 50% of families lived in regional areas. The mean age of mothers and fathers was 39 ± 7 and 44 ± 7 years, respectively; the mean age of siblings (5 girls, 4 boys) was 13 ± 4 years. The mean interview duration was 46 ± 12 minutes for parents and 15 ± 5 minutes for siblings. While data informing the final key parent themes identified in this study were collected in the first six interviews, all transcripts were coded to ensure maximum variation and to reach informational saturation. Key sibling themes were identified after coding all transcripts, although informational saturation was not reached.

**Table 1:** Sample characteristics.

Characteristics	*n* (%)
Child [*N* = 16]	
Age at parent interview, mean ± SD, years	6 ± 3
Male sex	12 (75)
Prenatal congenital heart disease diagnosis	9 (56)
Transposition of the great arteries/intact ventricular septum	15 (94)
Gestational age at birth, mean ± SD, weeks	39 ± 1
Age at arterial switch operation, mean ± SD, days	10 ± 4
Total length of hospital stay, median [IQR], days	19 [11]
Surgical re-intervention post discharge	0 (0)
Neurodevelopmental delay or disorder	2 (13)
Currently accessing developmental or schooling support	4 (25)
Total siblings, mean ± SD	2 ± 2
Total siblings living in the home, mean ± SD	2 ± 1
Extended family member/s living in the home	4 (25)
Australian Bureau of Statistics residential remoteness	
Urban	8 (50)
Regional	8 (50)
Parents [*N* = 28; 16 mothers, 12 fathers]	
Interview location	
Virtual	19 (68)
Face to face – Home	6 (21)
Face to face – Research Center	3 (11)
Age at interview, mean ± SD, years	
Mother	39 ± 7
Father	44 ± 7
High school graduate or below	
Mother	4 (25)
Father	4 (33)
Not currently in paid employment	
Mother	4 (25)
Father	1 (8)
Single parenthood	1 (7)
Identify as First Peoples of Australia	0 (0)
Country of birth	
Australia	17 (61)
Other – high income (New Zealand, United Kingdom)	4 (14)
Other – upper middle income (China, South Africa)	5 (18)
Other – lower middle income (India, Nigeria)	2 (7)
Siblings [*N* = 9; 5 sisters, 4 brothers from 7 families]	
Interview location	
Virtual	8 (89)
Face to face – Home	1 (11)
Age at interview, mean ± SD, years	13 ± 4
Current schooling	
University	2 (22)
High school	2 (22)
Primary school	5 (56)
Identify as First Peoples of Australia	0 (0)
Country of birth	
Australia	7 (78)
Other – upper middle income (South Africa)	2 (12)

### Key parent themes

**Fig. 2:**
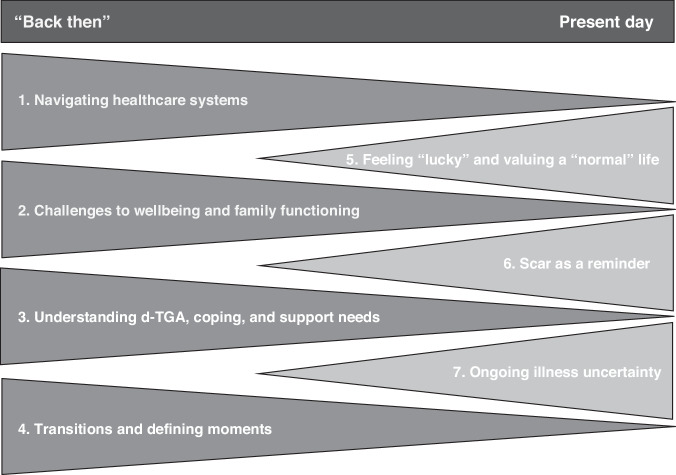
Key parent themes. This visual representation illustrates seven key parent themes, with themes 1-4 representing those experienced with greater frequency or intensity “back then” at the time of diagnosis and hospitalization but persist to the present day.

**Fig. 3:**
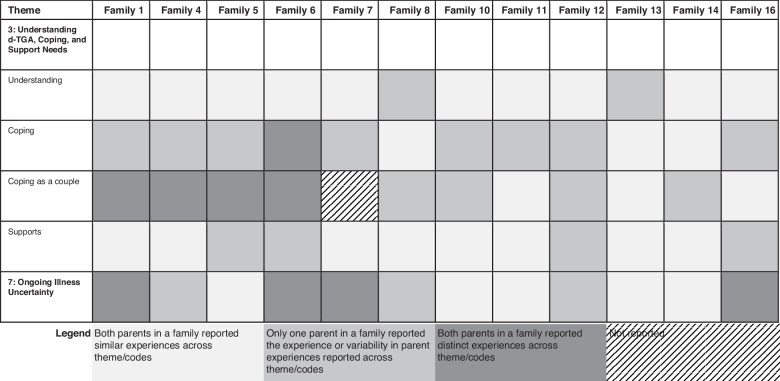
Comparisons of family members’ responses. This visual representation illustrates exemplar comparisons across themes/codes in two parent-participant families.

Figure 2 depicts seven key parent themes and exemplar quotes are shown in Table 2. Exemplar comparisons between responses of family members across themes/codes in two parent-participant families are illustrated in Fig. 3.

**Table 2:** Key parent and sibling quotes.

Themes	Key Quotes
Parent	
Theme 1: Navigating healthcare systems	“*Watching him go off and then pffft off he went in an ambulance. Like he’s just been born and he’s gone. And then you don’t, where’s he going?…Get to [hospital]. Park your car… trying to find emergency ward where he’d gone to. You had no idea*.” (Father 14, postnatal diagnosis, urban area) “*We always felt we, we were very fortunate that that we were here and that um, that you know, that our medical aid’s paid and, and that, that he had a brilliant surgeon and anesthetist and, and that people really cared about us and cared about him and took such great care of him you know. And like the skills of the nurses were amazing. Like the ICU nurses were absolutely outstanding and those like the heart nurses were just unbelievable with us and with him so*.” (Mother 14, postnatal diagnosis, urban area) “*You know but we, like I said, we’ve been through the medical system and we’ve tried to get some testing and we’ve spoken to people about behavioral issues and thing like that and they haven’t come to, they haven’t come through with testing and knowledge and stuff for us that we don’t know*.” (Father 13, antenatal diagnosis, regional area)
Theme 2: Challenges to wellbeing and family functioning	“*So…it was a bit of a, this sort of stuff doesn’t happen to me so it was a bit of a shock. And that first night when we found out in the afternoon and went home in the, went home, was probably the worst night of the entire experience. Um, but yeah it was a shock because I had never experienced anything like that before. Like it was sort of like that, this stuff doesn’t happen to me. This is what I see on the news or this is what I see happening to other people*.” (Father 11, antenatal diagnosis, regional area) “*But I sort of, I did feel a little, it was a big, it was massive. And then I remember coming home. And I was left on my own with this baby. Um, and I felt really alone. Um, but I guess it’s also because I didn’t have any family members you know or, or friends yet*.” (Mother 12, postnatal diagnosis, regional area) “*I think um no it’s still quite ah I think painful with seeing photos of him and things like that. Or remembering back to those times. I’d say they’ve lessened but they are definitely there in a strong sense still*.” (Mother 9, postnatal diagnosis, regional area)
Theme 3: Understanding d-TGA, coping, and support needs	“*You read the statistics and you get assurance from the specialist but that’s, that does only so much you know. I mean, TGA as I understand it, usually has quite a good long-term prognosis when you have the right treatment. Um, so we were assured by that but it was still, it was clearly very stressful*.” (Father 6, antenatal diagnosis, regional area) “*Once I got to learn about it and after his surgery I came to accept that my child’s got a heart condition and yeah*.” (Mother 7, postnatal diagnosis, regional area) “*But I think at the time I think I was in a lot of survival. And I had, there was so much happening and it all happened really fast that I just had to do it*.” (Mother 3, antenatal diagnosis, regional area) “*I don’t not talk about it, but I don’t, it’s not like I introduce myself and hey hi, I’ve had a child that’s had open heart surgery sort of thing. But I’m happy to talk about it when, if, um, if anyone ever asks me. You know, cos they can see his scar or um. You know, I’m happy to talk about it*.” (Mother 1, postnatal diagnosis, urban area) “*Look ah major events in life always happen. You either you know get on with it or go backwards, sort of thing so just yeah. It’s always getting on with the situation*.” (Father 8, antenatal diagnosis, urban area) “*I think it’s more family. So I think we could have been anywhere in Australia and as long as we had our family and friends around that was supportive I think we would have gone through it anywhere. Yeah*.” (Mother 16, antenatal diagnosis, urban area) “*Once you leave the hospital, um, you’re sort of out on your own really*.” (Mother 3, antenatal diagnosis, regional area) “*But you feel like with transposition of the great arteries because the outcomes really relatively quite good, you feel, you know, that you shouldn’t complain. But at the same time you feel this (laughs) secret trauma from it… So I would love to know another mum that had it so you could be like oh, you know, yeah*.” (Mother 6, antenatal diagnosis, regional area)
Theme 4: Transitions and defining moments	“*The goal of her first birthday. I think after that I felt, uh, a sense of relief…Yes…I think after those, that traumatic time, I’ve made peace with it*.” (Mother 12, postnatal diagnosis, regional area) “*And just seeing his development, how he’s able to do things that I worried about before. Especially in terms of soccer and running and fitness…but now it’s like oh, you just let him go*.” (Mother 3, antenatal diagnosis, regional area) “*I would probably say the last visit. It was like, you know, fifth or sixth visit. We’re like ok, you’re saying the same thing again…It’s that repeated reassurance that yes ok, he’s normal*.” (Father 11, antenatal diagnosis, regional area)
Theme 5: Feeling “lucky” and valuing a “normal” life	“*We were really quite lucky. Even in terms of heart conditions…the time in the hospital wards of seeing children with half a heart and those…children who have to have operation after operation. Knowing that despite the trauma he went through, he was one of the lucky ones to have survived*.” (Mother 9, postnatal diagnosis, regional area) “*And we’ve been really lucky so far. We really had no dramas along the way. It’s been relatively straight forward for something that is not straightforward at all*.” (Mother 4, antenatal diagnosis, urban area) “*Congenital heart disease lives with us. So we don’t live with it. It comes along on our ride. Does that make sense? Like it’s not dictating to us the path that we’re taking…It’s certainly things that we are mindful of first and foremost like, you know, we’re having to deal with it but. Life presents too many other challenges for that to be at the center point*.” (Mother 13, antenatal diagnosis, regional area) “*They’re not different to other people, they’ve just had an experience that other people haven’t had. But you’ve gotta treat ‘em the same as you do your other kids. Don’t make them feel different*.” (Father 13, antenatal diagnosis, regional area) “*The vast majority of our experience is just a lot of gratitude for actually living in a place where we can get the treatment. And having a healthy young boy…every day I think about it and feel very very grateful. That’s probably the key thing*.” (Father 6, antenatal diagnosis, regional area) “*You come through it and you do feel, like you grow from it. Like I feel like a different person in a good way than beforehand. I feel like I’ve got more layers to me and more life experience*.” (Mother 6, antenatal diagnosis, regional area) “*As time goes on, the more you deal with things the better you can become at them. You know, if another situation were to arise, now that we’re a stronger family unit and have been through that, how we deal with it next, something next time is, potentially is going to be a bit better*.” (Mother 5, postnatal diagnosis, regional area)
Theme 6: Scar as a reminder	“*I see the scar on his chest and that’s a pretty powerful reminder of what he’s been through*.” (Father 6, antenatal diagnosis, regional area) “*You always remember. And, and it always brings back um, the feelings that you felt. All the time, every time that I see it. And, you know, it just makes me love him a bit more, he’s a bit more special*.” (Father 8, antenatal diagnosis, urban area)
Theme 7: Ongoing illness uncertainty	“*Yeah I am very conscious, he’s a very normal and active child, and I’m, but I’m very conscious that, it does play at the back of my mind that I might, you know, I could lose him. You know, any time really. Anything could happen. And I know that anything could happen to anyone. You know, all of us could, you know, have an accident*.” (Mother 1, postnatal diagnosis, urban area) “*So like worst case scenario, it would mean further surgery in the future but yeah, we’ll tackle that if that happens when we get to it*.” (Mother 4, antenatal diagnosis, urban area)
Sibling	
Theme 1: “Normal” family life	“*Um, we have a good family. We all look after each other, care for each other, help each other out a lot. Kind of like a normal family*.” (Sibling 13b, antenatal diagnosis, regional area)
Theme 2: Getting along… but they can be annoying	“*We get on well. I would say that. Um like knowing other people’s siblings’ relationships I’d say we get on really well*.” (Sibling 14, postnatal diagnosis, urban area) “*It’s normal having a brother like that cos all together, I don’t like brothers*.” (Sibling 11a, antenatal diagnosis, regional area)
Theme 3: Understanding and experiencing my sibling’s CHD	“*It’s not that different. I mean for me it’s kind of like just having another sister. It doesn’t really change much. Other than the fact that she goes to appointments a couple of times a year that’s about it*.” (Sibling 13b, antenatal diagnosis, regional area) “*He has a scar on…his chest. And that he had an operation when he was born because of it. That’s all I know.”* (Sibling 13a, antenatal diagnosis, regional area) “*So the doctors usually check on him every year or month. That’s all I know…Sometimes I feel scared like there might, something might go wrong or if…I don’t know really*.” (Sibling 8, antenatal diagnosis, urban area)

#### Theme 1: Navigating healthcare systems

Parents described challenges navigating healthcare systems from the time of d-TGA diagnosis. Parents of infants diagnosed after birth described experiences of their infant becoming acutely unwell, with sudden separation from them when retrieved to the cardiac center. Parents recalled negative hospitalization experiences, including extended stays due to complications and gaps in physical and emotional care for new mothers in the transition from maternity to cardiac services.

For many parents, attending their child’s annual or biennial follow-up cardiology reviews caused minimal difficulties. Within families, mothers were more likely to experience worry at reviews. Almost half of the families interviewed were accessing developmental services or considering/trying to access assessment or intervention for their child. Of those accessing services, parents from three families identified ongoing concerns and/or additional services needed.

#### Theme 2: Challenges to wellbeing and family functioning

Irrespective of the d-TGA diagnosis timing, both mothers and fathers described shock, and subsequent fear and devastation. For many, this distress continued throughout hospitalization, especially when witnessing the care of their unwell infant and needing to make difficult decisions about their child’s medical care. Several mothers described feeling vulnerable as new mothers, their grief, and concerns for mental health. Within families, both mothers and fathers reported being concerned about siblings’ needs being met.

Into early childhood and the present day, the decreased impact of CHD on both the individual parent and family in comparison to “back then” was consistently identified. However, some mothers described ongoing personal guilt, worry, and fears, including concerning the potential sudden loss of their child. While most parents felt there were no ongoing negative effects on siblings, both parents in one family described challenges in sibling responses to stressful situations, and both parents in another family described the impact of behavioral challenges of the child with CHD on siblings and broader family life. Despite early relationship/marriage challenges during hospitalization, several parents now described their experiences as strengthening their relationship.

#### Theme 3: Understanding d-TGA, coping, and support needs

Almost all parents now have an understanding of their child’s heart condition which they describe in varying levels of detail. Regardless of diagnosis timing, clear, simple communication and the use of diagrams were highly effective in helping parents’ understanding. Several parents described the benefits of seeking additional online information and talking to family and friends with a medical background. Cardiology reviews are now a time of reassurance regarding the stability of their child’s condition, with the potential for future re-intervention identified for some.

Parents described their coping with their child’s heart condition as “getting on with” what they needed to do, especially at diagnosis and during hospitalization. Being organized, optimistic, easy-going, and solution-focused were identified as helpful, especially among fathers. Differences in maternal and paternal coping contributed to relationship strain, as well as being complementary and enabling them to function. Where relationships were strained, both mothers and fathers attributed this to the father distancing themselves from the hospital as a way of coping.

Several parents, predominantly mothers, identified the need for psychological support post-hospital discharge, which was not always accessed due to busyness, not feeling ready, or lack of knowledge or availability. A storybook created by one mother to process her experience became a valuable resource for all family members.

The closeness and emotional and practical support of extended family were crucial for families during hospital admission and transition home. Lack of extended family support, through relationship breakdowns or geographical distance, was keenly felt by both mothers and fathers. The importance of close friendships in providing support during hospitalization, as well as in current day-to-day life, was also identified. Some parents also described the positive role of other families affected by CHD and their local community. Formal psychosocial supports offered to parents during their hospital stay were not perceived to be needed by mothers or fathers, especially when well supported by family and friends. Where several parents identified the benefit of formalized peer support and advocacy groups, most did not engage beyond occasional social media access.

#### Theme 4: Transitions and defining moments

For some parents, reassurance from their child’s cardiac care team at hospital discharge or subsequent visits and reduced frequency of cardiology follow-up were key to moving forward with life. Positive experiences of their child’s development, including reaching milestones, starting school, and sporting achievements, also facilitated knowing that their child was progressing well. Among families that described a shift “back to normal”, the timing of this varied. For example, in one family, both parents felt life went back to normal soon after discharge, while in other families, this did not occur until one or two years post-discharge.

#### Theme 5: Feeling “lucky” and valuing a “normal” life

Feeling “lucky” regarding their child’s cardiac condition in relation to more complex conditions and modern cardiac care was a shared experience between and within families. Overall, parents identified feeling “lucky” for the place that CHD can hold in their lives and not be at the center of how they live.

Both mothers and fathers predominantly described themselves as a “normal” family, living a “normal” life with busy daily routines. They mostly described close relationships between their child with CHD and siblings, with some challenges due to differing personalities or during the sibling teenage years. Several parents described not seeing their child as having a heart condition and mostly seeing them as progressing as a typical child, with many positive individual qualities. Sibling comparison enabled parents to see similarities as well as developmental and behavioral differences between their children with and without CHD. Early over-protectiveness had eased for most parents, and many described the importance of treating their children just like other children. A few parents across different families described their child as “special” in the context of additional developmental differences, and one due to the cardiac condition.

Parents frequently described deep gratitude for the survival and health of their child, and to be living in Australia, with universal access to healthcare and resources. Many mothers and fathers had a strong awareness of and empathy for families who traveled extensive distances for clinical care, families of children requiring multiple surgeries, and bereaved families. Areas of personal growth, including a greater appreciation for life and compassion towards others, stronger family relationships, and resilience when challenges arise, were also identified between and within families.

#### Theme 6: Scar as a reminder

Although many parents described not seeing their child as having a heart condition, the surgical scar served as a constant reminder of what they and their child had experienced, as well as their child’s survival and bravery. While the potential for future bullying was identified, the scar was also seen as an opportunity to increase awareness of CHD.

#### Theme 7: Ongoing illness uncertainty

Some parents voiced ongoing illness uncertainty, including fears about their child’s future health and wellbeing, neurocognitive difficulties, and physical activity limitations. Parents were also aware of the uncertainty of future surgery, with some managing their worries by “dealing with it when it happens”. Within families, worry and anxiety related to ongoing uncertainties, including the need for future surgery, were more likely to be experienced by mothers, although greater worry or overprotectiveness was identified by two fathers.

### Sibling themes

Three themes were identified in the siblings’ narratives (see Table 2).

#### Theme 1: “Normal” family life

Consistent with parents, siblings described busy daily routines, including school/university, time with family and friends, extracurricular activities, and personal interests, contributing to a sense of “normal” life.

#### Theme 2: Getting along… but they can be annoying

Positive and negative aspects of the sibling relationship were identified, from spending time together and getting along well, to finding them frustrating and annoying, with just one sibling identifying this as a point of difference to other sibling relationships. Several siblings identified enjoying looking after and helping their younger sibling with CHD or feeling empathy when considering their cardiac condition.

#### Theme 3: Understanding and experiencing my sibling’s CHD

Consistent with their parents, most siblings also saw their sibling with d-TGA as “normal” and no different from other siblings. Differences identified were in the context of the sibling relationship (Theme 2), and the child with CHD being the only one in the family born with “something wrong”. Most thought of their sibling’s cardiac condition infrequently, with the surgical scar a reminder for some. Few could recall details of their sibling’s heart condition, and those who could recall the cardiac hospital admission identified it being a time of greater impact on their family than now. One sibling reflected on how her own (non-CHD related) hospitalization helped her understand the difficulty her parents experienced with “their little baby going through so much”. Although most siblings had minimal awareness of or worry about their sibling’s cardiology follow-up visits, one described feeling scared that “something might go wrong”.

## Discussion

In this qualitative study including parents and siblings of children with d-TGA, participants predominantly described positive adaptation and living and valuing their “normal” family lives, despite some ongoing uncertainties and reminders of their early experiences. Findings align with Rolland’s Family Systems – Illness Model^34,35^ identified previously among fathers of children with CHD.^36^ Families in our study have moved beyond the crisis phase of diagnosis and surgery, having achieved an understanding of their child’s condition, positive adaptation within the hospital environment, and maintaining hope while acknowledging potential future challenges.

Normalcy within the family system is subjective, culturally constructed, and does not imply the absence of challenges.^37^ The importance of a sense of normality has been identified in a systematic review of qualitative studies including families of children with CHD.^25,38,39^ “Chronic paradoxes” experienced by parents and families with a child with CHD have been described,^39^ where living a “normal” life is seen as vital but also a source of stress, particularly in the transition home from the hospital. Mothers and fathers in our study have predominantly established what they describe as “normal” family life, where they and others mostly treat their child “normally”, which may reflect the older age of children in this sample. The importance of treating your child “normally” has been reported in other studies of children with CHD.^25^ In our study, two facilitating factors were identified. First, through reassurance and encouragement by treating clinicians, consistent with earlier studies of parents of children with d-TGA, where parents were encouraged to treat their child as “normal” and considered “fixed”.^9,10^ Second, parents’ own experiences of seeing their child’s progress and achievements, consistent with the experience of parents of children with single ventricle conditions.^26^

Results from our study are consistent with others examining families of children and adolescents with CHD, where both predominantly no difference between parent responses has been reported,^36,40,41^ as well as greater psychological distress among mothers than fathers.^40,42,43^ While mothers and fathers in the current study predominantly described not living a life focused on their child’s CHD, some mothers described ongoing experiences of guilt, worry, and fear. Parents also identified positive outcomes through personal growth and strengthening of relationships within the family system. Increased compassion, strength in coping, and appreciation for the family have been reported by parents of children with HLHS,^44^ with greater family closeness identified in other studies of children with childhood heart conditions,^21^ including families of children with d-TGA.^9^ Studies of post-traumatic growth and individual resilience in CHD family populations are very limited, as are longitudinal studies of family resilience more broadly.^45^ Such studies may be valuable in understanding the experiences of, and protective factors for families of children with complex CHD, particularly at times of key clinical and social transitions and where future uncertainty is evident.^9,46^

To our knowledge, our study is the first to report on parental and sibling perceptions of the surgical scars of their child and sibling with CHD. Studies examining scar perceptions of adolescents and adults with CHD themselves are also limited and, consistent with our findings, identify both negative (impact on self-image)^47^ and positive (appreciation of their health) effects.^48^ Those with greater disease complexity were more affected than those with simple conditions,^48^ however, parental perception of scarring across disease complexity is wholly unknown. Furthermore, optimal strategies for communicating with and supporting parents and siblings regarding their child’s and sibling’s scars are also unknown.

Consistent with recent reviews,^49,50^ most siblings in our study did not identify being affected in daily life by their sibling’s CHD, likely due to the good health of the child with CHD and lack of CHD-related caregiving needs. Negative feelings such as jealousy and resentfulness identified in studies of siblings of children with CHD^21^ and other conditions^51^ were also not described. A lack of discussion of the condition by parents with siblings in our study is consistent with the experiences of siblings of children with other chronic illnesses;^52^ however, this may be due to a perceived lack of impact on the family as opposed to protecting siblings.

Strengths of the current study include exploration of the experiences of a cohort with a predominantly homogeneous clinical course, as well as the participation of multiple family members and subsequent response comparisons. Limitations of the study, however, are also noted. Our findings may not be generalizable to families of children with other cardiac conditions or those who do not have access to socialized medicine. Data saturation was not achieved for sibling interviews; however, given the importance of, but very limited, qualitative evidence of sibling experiences, the current study makes an important contribution.

In conclusion, while parents and siblings of children with d-TGA value living “normal” family lives, opportunities exist for improved care and future research. Persisting distress for some parents and variability in transition to a sense of normality highlight the need for ongoing communication with families regarding their wellbeing including at routine cardiac review. Our study supports the identified need for further exploration of positive processes and outcomes, including individual and family resilience, across time. Further understanding of family members’ perceptions of surgical scars is also needed to ensure information and support of families is inclusive of their own experience, as well as that of the child with CHD. Finally, self-reported sibling experiences are needed to better understand and address education and support needs over time.

## Supplementary information

Eagleson et al_Pediatric Research_Final Supplement_June 2025

## Data Availability

Datasets generated and/or analyzed for this study will be available on reasonable request from the corresponding author.
