# Supplementary material for: Longer-term experiences of families of children with dextro-transposition of the great arteries: a qualitative study
Source: Pediatr Res. 2025 Jul 17;99(7):2624–33. doi: 10.1038/s41390-025-04201-y (PMC13485675; doi:10.1038/s41390-025-04201-y)
Supplement: Supplementary file 1 [file 41390_2025_4201_MOESM1_ESM.pdf]

List of Supplementary Materials

Supplement 1: Interview Guide

Supplement 2: Example of Coding Cycles and Theme Development

Supplement 1: Interview Guide

Parent:

1. **Can you tell me about you and your family?**

   Prompts:

   - Consider identifying extended family members who may be important to the immediate family.

2. **What was your experience of learning about (name of child with TGA) heart condition?**

   Prompts:

   - Did you learn about your child's heart condition before they were born?
   - Where were you when you learned about your child's heart condition?

3. **Can you tell me about your understanding of (name of child with TGA) heart condition?**

   Prompts:

   - Can you describe the care that your child has needed in the past?
   - Can you describe the care that they need now and into the future?

4. **Can you tell me about family life before you had (name of child with TGA)?**

   Prompts:

   - Can you describe other important life events you have experienced as a family?

5. **How has (name of child with TGA) heart condition affected/impacted <u>you personally</u>?**

   Prompts:

   - Can you describe to me the experiences that have impacted you the most?
   - How has (name of child with TGA) heart condition impacted you changed over time?

6. **How do you think (name of child with TGA) heart condition has affected/impacted your <u>family as a whole</u>?**

   Prompts:

   - Can you describe to me the experiences that have impacted your family the most?
   - How has (name of child with TGA) heart condition impacted your family changed over time?
   - What have been the greatest challenges for you and your family?
   - What are the greatest unmet needs for you and your family?
   - Have there been positive experiences or outcomes for you and your family?

7. **Can you describe to me how <u>you and your family</u> have coped with (name of child with TGA) heart condition?**

Prompts:

- Can you describe to me who or what has been available to you/your family to help you cope?
- In coping with (name of child with TGA) heart condition, can you describe to me what has worked the best for you and your family?
- Can you describe to me something that you tried or was offered to you that did not help your coping?
- How do you think where you live has impacted how you and your family cope?
- **Note if the focus is more on the individual or family.

8. **Can you describe life for <u>you and your family</u> now, living with CHD?**

Prompts:

- Can you describe any positive changes that have occurred since having (name of child with TGA)? (May be a prompt if only negative/neutral identified).
- Can you describe any negative changes that have occurred since having (name of child with TGA)? (May be a prompt if only positive/neutral identified).
- **Note if the focus is more on the individual or family.

9. **What advice would you give other families living with CHD?**

Siblings (age 8 years and over):

1. **Can you tell me about you and your family?**

Prompts:

- Who is in your family?
- What do you like to do?
- What does your family like to do?

2. **Can you tell me about (name of brother/sister with TGA) heart condition?**

Prompts:

- Do you know the name of their heart condition?
- How did you learn about their heart condition?
- Do you know what happens when your brother/sister visits the heart doctor?

3. **Can you tell me/describe to me what it's like having (name of brother/sister with TGA) as your brother/sister?**

Prompts:

- Can you tell me about your relationship with (name of brother/sister with TGA)?

- Can you tell me about (name of brother/sister with TGA) relationship with other people in the family?

4. **What is the best part of being (name of brother/sister with TGA) brother/sister?**

   Prompts:

   - What do you like about them?
   - What do you like to do together?

5. **What are the hardest parts of being (name of brother/sister with TGA) brother/sister?**

   Prompts:

   - What are the things you do not like about being (name of brother/sister with TGA) brother/sister?

6. **What helps you handle the tough parts?**

   Prompts:

   - What do you do if you are having a tough time with your brother/sister?
   - Do you talk to someone? Who do you talk to?

7. **What advice would you give other kids who have a brother/sister with CHD?**

   Prompts:

   - What do you think might be helpful for other children with a sibling with CHD to know?

Supplement 2: Example of Coding Cycles and Theme Development

<table>
<tr><th>First Cycle</th><th>Second Cycle</th><th>Third Cycle / Theme</th></tr>
<tr><td>Stronger family
Life is easier now
Appreciating your family
Minimal impact of heart condition on child/family/couple</td><td>Positive Outcomes</td><td rowspan="3">“Feeling lucky” and valuing a “normal” life</td></tr>
<tr><td>Gratitude
Appreciating your family
Relief (in response to child’s condition)</td><td>Gratitude</td></tr>
<tr><td>“Normal” family
“It doesn’t affect us anymore”
“Live a normal life”
Not seeing child as having a heart condition</td><td>Normal Expectations</td></tr>
</table>
